# Annotations

**Published:** 1904-09-24

**Authors:** 


### Annotations.

### "Remitting the Surgeon's Fee."

The proposal of one of the large London hospitals to institute a system by which the charitable funds of the hospital may be protected from persons who have no proper claim to such assistance has led to various activities in the daily press and has produced a mild sensation in the dead season. Besides the usual effusions from anonymous and irresponsible correspondents there have appeared reports of certain interviews with persons who are vaguely defined as "officials." In one of these there appears the phrase placed at the head of this note and the circumstances in which it was used are somewhat significant. The dignitary in question informs the interviewer that "we" have a home for paying patients where for three guineas a week, board, lodging, and attendance are provided for well-to-do persons, the surgeon's fee and medical treatment in such cases being extras. But if the patient has not a long purse there is, it appears, an opportunity for a display of thoughtful consideration of which "we" in a spirit of benevolence take full advantage. The benevolence, however, is a vicarious performance, and it consists in "remitting the surgeon's fees altogether." By those ignorant of the art of charity it might have been deemed fitting that both "we" and the surgeon should have agreed to reduce their respective charges, or that, in any event, the surgeon should have been credited with the entire value of his self-denial. But these expectations are only for the ingenuous. The blaze of benevolence which is now flashed upon the world permits no such result. On the contrary, "we," triumphing in his charitable plan, may be seen collecting his usual charges, whilst for the surgeon there exists a committee by whom his fees are "remitted altogether." This picture of graded or organised charity is a very touching one, and no wonder the gentleman responsible for it contemplates it with modest satisfaction. To be charitable oneself is well enough in its way, but to be the cause of charity in others is indeed a triumph.

### Brompton Hospital Report.

The issue of a report on the work of the pathological department of the Brompton Hospital is an event which deserves appreciative notice, all the more so that the pamphlet can be obtained for the modest charge of one shilling. On more than one occasion we have urged that the duty of those who enjoy the great opportunities associated with hospital appointments is not limited to the care of individual patients but includes the spread of information among the profession generally. Therefore it is with sincere satisfaction that we have received a carefully-compiled series of tables which display the facts observed in the post-mortem room of the leading hospital for diseases of the chest in this country. The motive which has led to the issue of the report ought to command general appreciation. There is hardly any aspect of medical development of the absence of which the general practitioner is more conscious than his inability to continue his acquaintance with post-mortem facts. And this refers, not to the refinements of the laboratory, but to the coarse changes to be seen on dissection. The authors of the Brompton report have been well advised to present facts of this order rather than to describe details only accessible to special methods of microscopic examination. Still more valuable are the tables in which the broad facts found on post-mortem examination are associated with a short but succinct description of the clinical aspects of the various cases. The analyses of the cases of tuberculous pneumothorax and of malignant disease of the lung are particularly serviceable in this respect. The details, however, are less our text here than the actual issue of the report. That fact in itself as well as the plan of arrangement and style of issue all show a desire to spread the lessons to be learned from this great hospital over a wide area. It is pleasant to note the existence of this spirit and to acknowledge gratefully the form and manner in which it has expressed itself. If we venture to hint that the plan may be further developed the lively sense of favours to come is not inconsistent with true gratitude.

### The Conscientious Objector and the Increase of Vaccination.

It is probable that the opponents of compulsory vaccination will cite the facts and figures given in the report of the Medical Officer of the Local Government Board for 1902–3, which was published on Tuesday, as a reason for the maintenance of the conscientious objector. His continued existence certainly does not, we are glad to admit, prevent the increasing observance of the Vaccination Act of 1898. The report before us is on this point emphatically reassuring. In England the percentage of certificates of successful vaccination has increased in every county, the increase ranging from 7·0 in Bedfordshire to 0·1 in the North Riding. It is true that the certificates of conscientious objection have increased in a considerable number of counties, but in the majority there has been a decrease, as also in most of the Welsh counties. A more significant result is that in 1901 there was a definite increase of infant vaccination in every one of the 43 English counties, and in seven Welsh counties. Moreover, while the observed increase of vaccination ranges from 0·1 per cent. to 7 per cent., the increase of conscientious objection has nowhere exceeded 2·2 per cent., and every case of conscientious objection has been accompanied by a correspondingly greater increase of vaccination. Of course, we attribute the improvement in the position indicated to better administration on the part of the authorities, and, perhaps, in some degree to the steadily growing conviction, even amongst the most ignorant, that vaccination is an almost unfailing safeguard against small-pox. But the conscientious objector is none the less a distinct source of danger to the community, and we cannot therefore hold out to him any hope that because he is unable to hinder the officials of the Local Government Board from doing their duty, and the public from protecting themselves against the ravages of a terrible disease, he will be permitted to have free course.

